# UNC5B receptor deletion exacerbates DSS-induced colitis in mice by increasing epithelial cell apoptosis

**DOI:** 10.1111/jcmm.12280

**Published:** 2014-04-10

**Authors:** Punithavathi Ranganathan, Calpurnia Jayakumar, Dean Y Li, Ganesan Ramesh

**Affiliations:** a Vascular Biology Center, Georgia Regents UniversityAugusta, GA, USA; b Program In Molecular Medicine, University of UtahSalt Lake City, UT, USA

**Keywords:** DSS colitis, UNC5B, netrin-1, apoptosis, inflammatory bowel disease

## Abstract

The netrin-1 administration or overexpression is known to protect colon from acute colitis. However, the receptor that mediates netrin-1 protective activities in the colon during colitis remains unknown. We tested the hypothesis that UNC5B receptor is a critical mediator of protective function of netrin-1 in dextran sodium sulfate (DSS)-induced colitis using mice with partial deletion of UNC5B receptor. DSS colitis was performed in mice with partial genetic UNC5B deficiency (UNC5B^+/−^ mice) or wild-type mice to examine the role of endogenous UNC5B. These studies were supported by *in vitro* models of DSS-induced apoptosis in human colon epithelial cells. WT mice developed colitis in response to DSS feeding as indicated by reduction in bw, reduction in colon length and increase in colon weight. These changes were exacerbated in heterozygous UNC5B knockout mice treated with DSS. Periodic Acid-Schiff stained section shows damages in colon epithelium and mononuclear cell infiltration in WT mice, which was further increased in UNC5B heterozygous knockout mice. This was associated with large increase in inflammatory mediators such as cytokine and chemokine expression and extensive apoptosis of epithelial cells in heterozygous knockout mice as compared to WT mice. Overexpression of UNC5B human colon epithelial cells suppressed DSS-induced apoptosis and caspase-3 activity. Moreover, DSS induced large amount of netrin-1 and shRNA mediated knockdown of netrin-1 induction exacerbated DSS-induced epithelial cell apoptosis. Our results suggest that UNC5B is a critical mediator of cell survival in response to stress in colon.

## Introduction

Inflammatory bowel diseases (IBD) such as ulcerative colitis and Crohn's disease are immune and inflammatory disease of the gastrointestinal tract. Faulty immune system attacks the cells that lining of the intestines where they produce chronic inflammation. Apoptosis and destruction of protective epithelium causes barrier breakdown that may require surgery. The worldwide incidence rate of ulcerative colitis varies considerably between 0.5 and 24.5/100,000 persons, while that of Crohn's disease varies between 0.1 and 16/100,000 persons worldwide, with the prevalence rate of IBD reaching up to 396/100,000 persons [1]. It is estimated that as many as 1.4 million individuals in the United States suffer from these diseases. Currently, there are no effective treatments available to treat IBD. Recent studies had determined that either administration of recombinant netrin-1 [2] or overexpression in colon epithelial cells [3] suppressed dextran sodium sulfate (DSS) colitis, reduced epithelial cell apoptosis and inflammation. However, the receptor that may mediate netrin-1 protective role in colon epithelial cells is not clear. Netrin-1 is primarily known as a chemotropic factor that attracts or repels axons depending on which netrin receptor is expressed on the individual axon [4,5]. Netrin-1 and its receptors, deleted in colorectal cancer and uncoordinated gene 5H (which includes UNC5H1-4/UNC5A-D) are highly expressed in the colon epithelial cells as well as immune cells [3,6,7]. Deleted in colorectal cancer and UNC5H are known as dependence receptors as they send either survival or apoptotic signals to cells dependent on the presence or absence of their ligand, netrin-1 [8,9]. However, dependence receptor hypothesis is not entirely validated. For example, kidney epithelial cells are known to express very little or no netrin-1 under basal condition, but UNC5B receptor is constitutively expressed but still not much apoptosis was seen in normal kidney. It is possible that ligand dependence may be tissue specific or UNC5B receptor may play a critical role in cell survival under stress. In the present study, we investigated the hypothesis that UNC5B receptor may be a critical mediator of cell survival in response to injury using UNC5B heterozygous knockout mice in a model of DSS colitis.

## Materials and methods

### DSS colitis

The Institutional Animal Care and Use Committee of the Georgia Health Sciences University approved all of the protocols and procedures using animals (approval number-2011-0348). Eight-week-old wild-type or heterozygous knockout mice for UNC5B receptor were used in DSS studies. UNC5B heterozygous knockout mice were created as described previously [10]. For details about DSS-induced colitis model, please refer to the following reviews [11,12]. A percentage of 3.5% DSS (MP Biomedicals, Santa Ana, CA, USA) were added in drinking water. The DSS was removed after 8 days and then animals were fed with normal water. Animals were killed on Day 2 after removal of DSS. Colon tissues were collected and processed for histology and RNA isolation. Colon was flushed with PBS to remove faecal materials. Then wet weight and length of the colon was measured.

### Quantification of mRNA by real-time RT-PCR

Real-time RT-PCR was performed in an Applied Biosystems Inc. 7700 Sequence Detection System (Foster City, CA, USA). Total RNA of 1.5 μg was reverse transcribed in a reaction volume of 20 μl using Omniscript RT kit and random primers. The product was diluted to a volume of 150 μl, and 6 μl aliquots were used as templates for amplification using the SYBR Green PCR amplification reagent (Qiagen Inc., Valencia, CA, USA) and gene-specific primers. The primer sets used were as follows: mouse tumour necrosis factor-α (forward: GCATGATCCGCGACGTGGAA; reverse: AGATCCATGCCGTTG GCCAG), monocyte chemotactic protein-1 (MCP-1) (forward: ATGCAGGTCCCTGTCATG; reverse: GCTTGAGGTGGTTGTGGA), intercellular adhesion molecule-1 (ICAM-1) (forward: AGATCACATTCACGGTGCTG; reverse: CTTCAGAGGCAGGAAACAGG), Netrin-1 (forward: AAGCCTATCACCCACCGGAAG; reverse: GCGCCACAGGAATCTTGATGC), osteopontin (forward: TCACCATTCGGATGAGTCTG-3′; reverse: ACTTGTGGCTCTGATGTTCC), interleukin-6 (forward: GATGCTACCAAACTGGATATAATC; reverse: GGTCCTTAGCCACTCCTTCTGTG), interferon gamma-induced protein 10 (IP-10) (forward: GGTCTGAGTGGGACTCAAGG; reverse: CGTGGCAATGATCTCAACAC) and transforming growth factor-β1 (forward: TGACGTCACTGGAGTTGTACGG; reverse: GGTTCATGTCATGGATGGTGC). The amount of DNA was normalized to the β-actin signal amplified in a separate reaction (forward primer: AGAGGGAAATCGTGCGTGAC; reverse: CAATAGTGATGACCTGGCCGT). Pro-apoptotic gene expression was quantified using apoptosis PCR array (Realtimeprimers.com).

### TACS TdT *in situ* apoptosis detection

To identify apoptotic cells, tissue sections were stained using TACS TdT *in situ* Apoptosis Detection kit (R&D Systems Inc., Minneapolis, MN, USA) according to the manufacturer's instruction. Briefly, tissue sections were deparaffinized, hydrated and washed with PBS. Sections were digested with proteinase K for 15 min. at 24°C. Slides were then washed and endogenous peroxidase activity was quenched with 3% H_2_O_2_ in methanol. Slides were washed and incubated with TdT labelling reaction mix at 37°C for 1 hr and then with streptavidin-horseradish peroxidase. Colour was developed using TACS blue label substrate solution. Slides were washed, counterstained and mounted with Permount. Sections were photographed and labelled cells were counted and quantified.

### Histology and immunostaining

Samples of proximal colon were fixed in 10% buffered formalin and stained with haematoxylin and eosin. The histological examination was performed in a blinded fashion using a scoring system previously validated and described [13]. Three independent parameters were measured: severity of inflammation (0–3: none, slight, moderate, severe), depth of injury (0–3: none, mucosal, mucosal and submucosal, transmural), crypt damage (0–4: none, basal 1/3 damaged, basal 2/3 damaged, only surface epithelium intact, entire crypt and epithelium lost) and percentage of the involved area (0–4: 0%, 1–10%, 10–25%, 25–50%, 50–100%). All scores on the individual parameters together could result in a total score ranging from 0 to 14. Stained sections were photographed using an Olympus inverted microscope with colour CCD camera (Center Valley, PA, USA). To quantify leucocyte infiltration, sections were stained with rat antimouse neutrophil antibody (Abcam, Cambridge, MA, USA; 1:200 dilution) or rat antimouse macrophage antibody (Catolog # ab56297; Abcam; 1:200 dilution) followed by goat anti-rat biotin conjugate.

To determine endogenous mouse netrin-1 and UNC5B protein expression, sections were stained with goat anti-netrin-1 polyclonal antibody (1:100 dilution; Santa Cruz Biotechnology Inc., Dallas, TX, USA) and rabbit anti-UNC5B polyclonal antibody (1:200 dilution; MD Millipore Corporation, Billerica, MA, USA) followed by secondary antibody conjugated with peroxidase polymer (Vector Laboratories, Burlingame, CA, USA). Colour was developed after incubation with ABC reagent (Vector Lab). Stained sections were photographed using an Olympus inverted microscope with colour CCD camera.

### Quantification of apoptosis by flow cytometry

To quantify the influence of UNC5B expression on DSS-induced cell death, human colon epithelial cells were transfected with 4 μg/well of rat UNC5B expression construct (Gift from Prof. Patrick Mehlen, Centre Leon Berard, Lyon, France) in a 6-well plate. Forty-eight hours after transfection, cells were treated with saline or different concentration of DSS. Cells were harvested at 24 hrs after treatment of DSS. To quantify apoptosis, cells were washed and stained for Annexin V-FITC and propidium iodide (Cat #640914; Biolegend, San Diego, CA, USA). Stained cells were immediately analysed by flow cytometry (BD FACSCalibur, BD biosciences, San Jose, CA, USA) and the data were analysed using Cyflogic V.1.2.1 software (CyFlo Ltd., Turku, Finland). Some harvested cells were used for determining netrin-1 expression and caspase 3 activity. Caspase-3 activity was quantified using an assay kit (BioAssay Systems, Hayward, CA, USA). Supernatant was used to determine the expression of netrin-1 in response to DSS treatment by Western blot analysis.

### UNC5B overexpression in TKPTS cells

To determine the effects of UNC5B overexpression on renal epithelial cell apoptosis, immortalized mouse kidney proximal tubule epithelial cells (TKPTS) cells were transfected with 2 μg/well of rat UNC5B expression construct (Gift from Prof. Patrick Mehlen, Centre Leon Berard) in a 6-well plate. Forty-eight hours after transfection, cells were treated with saline or different concentration of DSS.

### Knockdown of netrin-1 expression using shRNA

Human colon epithelial cells were transfected in a combination of four shRNA against netrin-1 expressing plasmid DNA (Sigma-Aldrich Corp., St. Louis, MO, USA). Seventy-two hours after transfection, cells were treated with different concentration (0.87, 1.75 and 3.5%) DSS or vehicle. Twenty-Four hours after addition of DSS, cells were harvested for quantification of apoptosis by flow cytometry as described above. Knockdown of netrin-1 was confirmed by RT-PCR and Western blot analysis.

### Statistical methods

All assays were performed in duplicate or triplicate. The data are reported as mean ± SEM. Statistical significance was assessed by an unpaired, two-tailed Student's *t*-test for single comparison or anova for multiple comparisons.

## Results

### UNC5B deletion abolishes DSS-induced netrin-1 expression in the colon

**Fig. 1:**
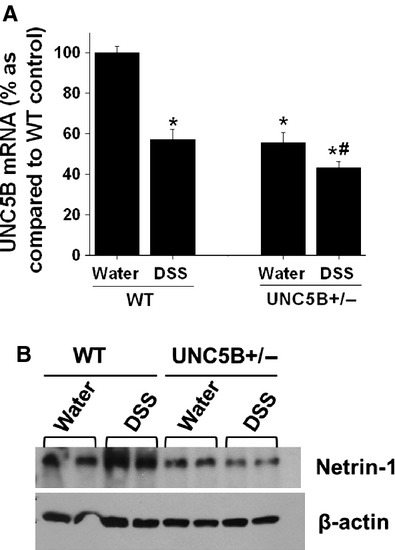
Regulation of UNC5B and netrin-1 expression in response to DSS treatment. (**A**) UNC5B expression was determined by RT-PCR. DSS treatment significantly reduced UNC5B expression. UNC5B^+/−^ mice shows 50% reduction expression under basal condition consistent with genotype. *P < 0.05 *versus* water-treated WT. ^#^*P* < 0.05 *versus* water-treated UNC5B^+/−^. (**B**) Netrin-1 expression in the colon was quantified by Western blot analysis. DSS induced netrin-1 expression as compared water-treated control in WT mice, but not in UNC5B^+/−^ mice.

To determine the role of UNC5B in DSS-induced colitis, we used mouse with one allele of UNC5B deleted. RT-PCR analysis showed a 50% reduction in expression of deleted animals as compared to WT animal colon (Fig. 1A). DSS treatment down-regulated UNC5B expression in both WT and UNC5B heterozygous knockout animal colon.

As UNC5B is known to mediate survival signal in the presence of netrin-1, the expression of netrin-1 was quantified by Western blot analysis. As shown in Figure 1B, DSS treatment was significantly up-regulated in WT mice colon which was blunted in UNC5B heterozygous knockout mice colon. This data suggest the presence of autoregulatory loop between receptor and its ligand.

### Partial deletion of UNC5B in mice exacerbates DSS colitis

**Fig. 2:**
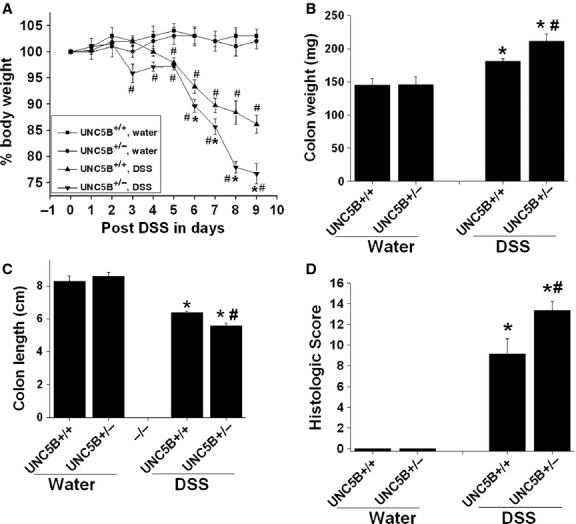
Partial deletion of UNC5B receptor exacerbates DSS colitis in mice. Disease activity in mice with partial deletion of UNC5B (heterozygous) and WT during DSS colitis. Gender-, age- and weight-matched heterozygous (partial deletion) mice and their wild-type (WT) littermates were exposed to DSS (3.5%) or water for 7 days, followed by sacrifice on Day 9 and harvesting of the whole colon by blunt dissection. (**A**) Daily weight measurements were obtained for each group of mice. ^#^*P* < 0.05 *versus* water treated. *P < 0.05 *versus* other groups. (**B**) At harvest, colon weight was measured for each mouse and is displayed as the mean ± SEM. *P < 0.001 *versus* water treated and #*P* < 0.05 *versus* other groups. *N* = 6–8. (**C**) Colon length at harvest was measured for each mouse and is displayed as mean ± SEM. *P < 0.001 *versus* water treated and ^#^*P* < 0.05 *versus* other groups. (**D**) Histological injury was scored as described in Methods. *P < 0.001 *versus* water treated. ^#^*P* < 0.05 *versus* DSS treated UNC5B^+/+^ (WT).

**Fig. 3:**
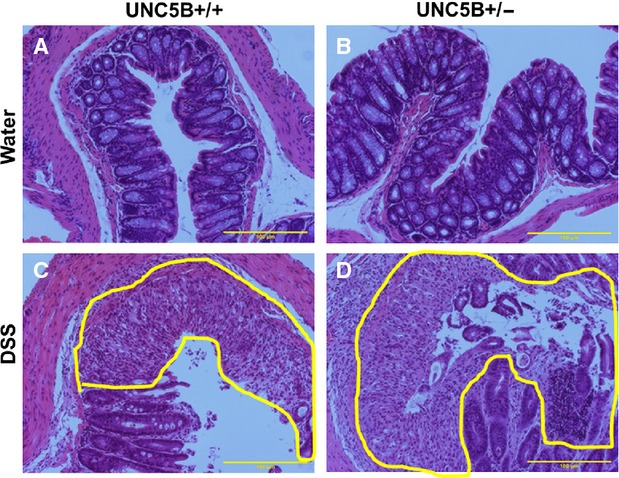
Periodic Acid-Schiff-stained colon section showing damage in gland, epithelium and infiltration of large number of white cells. Heterozygous mice treated with DSS show extensive damage as compared to WT mice with DSS. Damaged area is highlighted in yellow; *N* = 6–8.

**Fig. 4:**
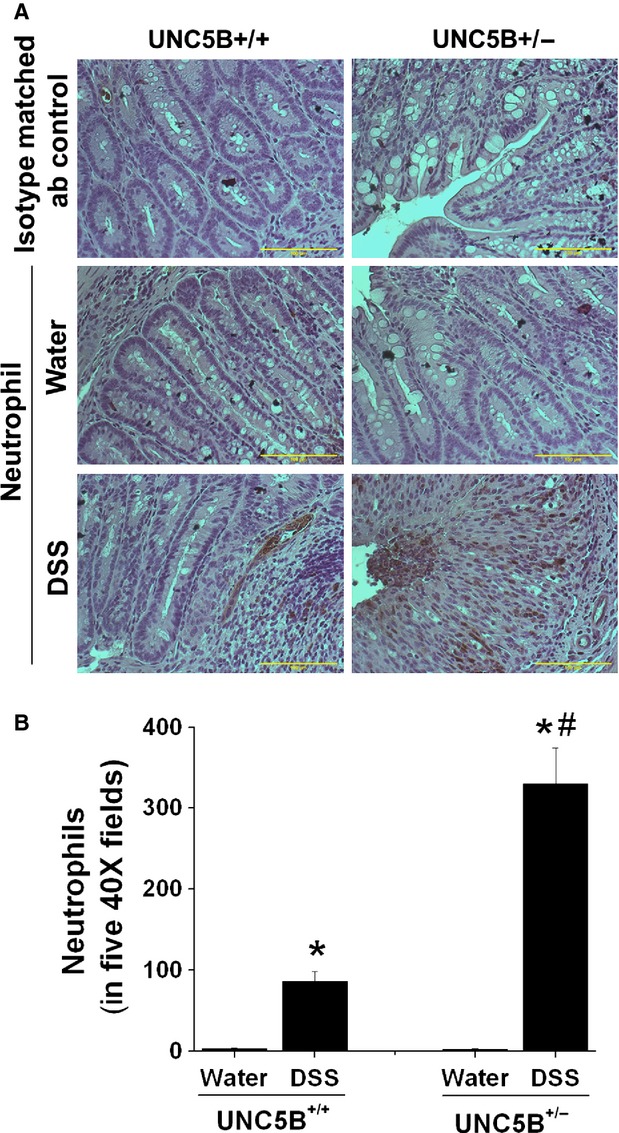
Immunohistochemical localization of neutrophils in WT and heterozygous UNC5B knockout mice colon that are fed with water or DSS. Neutrophil staining was carried out as described in Materials and methods. (**A**) No staining was seen with isotype matched antibody controls. Very few cells were positive neutrophils in water-treated UNC5B^+/+^ and UNC5B^+/−^ colon. DSS treatment increased neutrophils influx as seen by increased staining for neutrophils in colon mucosa. (**B**) Positive cells were quantified by counting stained cells in five 40 × fields in each animal colon. *P < 0.001 *versus* water treated. ^#^*P* < 0.001 *versus* DSS treated UNC5B^+/+^ mice; *n* = 4.

To determine whether UNC5B has the protective role in other models of tissue injury, we treated WT and heterozygous knockout mice with DSS as described in Methods. Body weight was monitored. Wild-type mice developed colitis as seen by a significant reduction in bw on Day 5 and reached the maximum of Day 9 when we killed. However, UNC5B^−/+^ mice developed much severe colitis as seen by significant loss of bw, increase in colon weight and reduction in colon length (Fig. 2A–D). Consistent with changes in bw and colon weight, histological examination shows severe tissue injury (Fig. 3A–D) and leucocyte infiltration (Fig. 4A and B and Fig. S2) in WT, which was further increased in UNC5B^−/+^ mice colon.

**Fig. 5:**
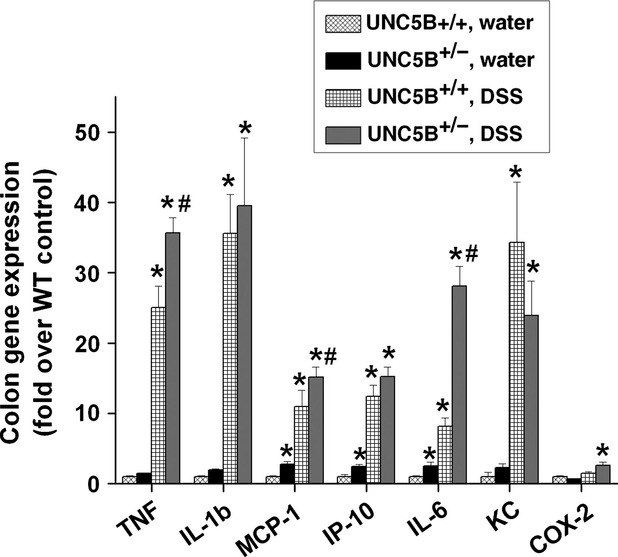
DSS treatment induced large number of cytokines and chemokine expression in WT, which was further increased in heterozygous mouse colon. Cytokines and chemokines expression was quantified by real-time RT-PCR as described before. *P < 0.001 *versus* Water treated. ^#^*P* < 0.05 *versus* DSS treated UNC5B^+/+^ (WT); *n* = 6.

**Fig. 6:**
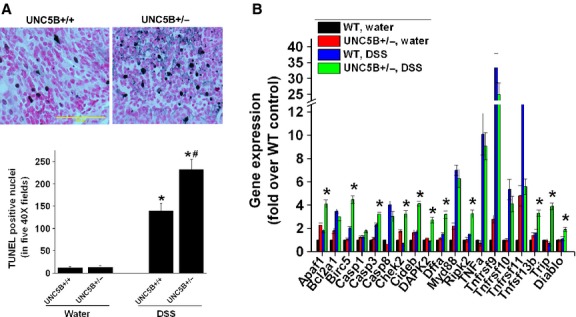
UNC5B deletion exacerbates epithelial cells apoptosis. (**A**) Apoptosis of colonic cell following DSS colitis in WT and UNC5B heterozygous knockout mice was determined by TUNEL assay. Quantitative data for TUNEL-positive nuclei in five 40 × fields are given below. DSS induced a large increase in apoptotic cells in WT mice, which was further increased in UNC5B heterozygous knockout mice. *P < 0.001 *versus* water treated and ^#^*P* < 0.05 *versus* other groups; *N* = 6–8; scale bar: 100 μM. (**B**) Pro-apoptotic gene expression was significantly increased in UNC5B^+/−^ with DSS as compared WT mice or water-treated control mice. *P < 0.001 *versus* other groups; *n* = 5.

Gene expression analysis showed that DSS induced a significant increase in the expression of inflammatory cytokines and chemokines in WT mice treated with DSS over water-treated mice, which was further increased in UNC5B^−/+^ mice treated with DSS (Fig. 5). Increased inflammation was associated with increased apoptosis in the colonic epithelial cells which was further increased in UNC5B^−/+^ mice treated with DSS (Fig. 6A). Consistent with increased apoptosis, the expression of several pro-apoptotic genes was significantly increased in UNC5B^+/+^ WT mice treated with DSS as compared to water-treated control, which was further increased in UNC5B^+/−^ mice treated with DSS (Fig. 6B).

### DSS treatment alters the compartmentalized expression of netrin-1 and UNC5B in the colon epithelial cells

**Fig. 7:**
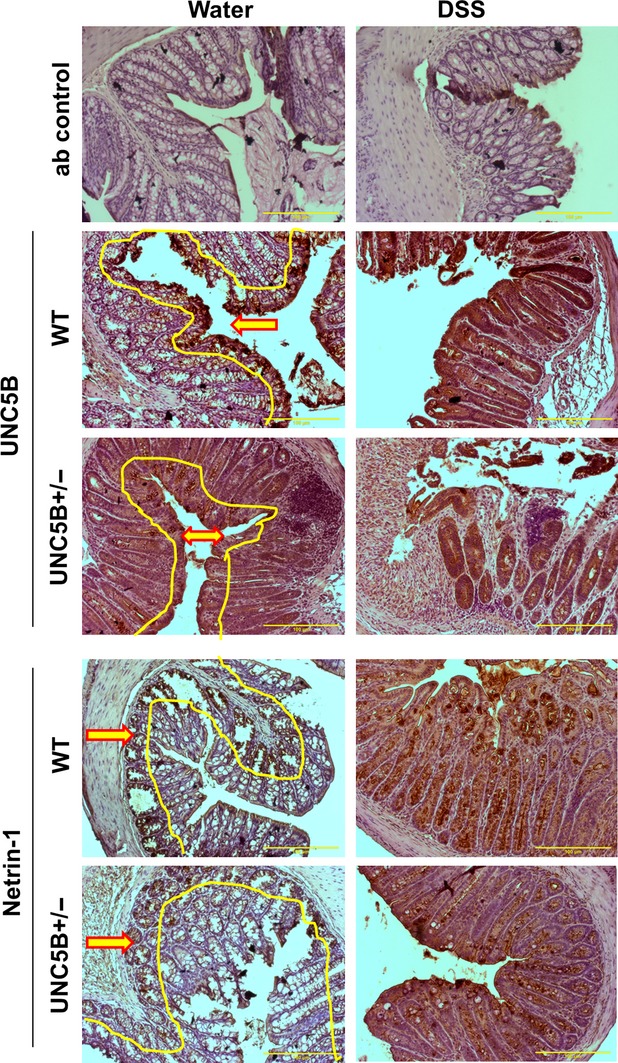
Immunhistochemical localization UNC5B receptor and netrin-1 in WT and UNC5B^+/−^ mice colon treated with water or DSS. No staining was seen in isotype-matched antibody control. UNC5B staining is restricted to apical epithelial cells of the mucosa (highlighted in yellow with arrow pointing towards apical surface). Intensity of staining is reduced in UNC5B^+/−^ mice colon. DSS treatment induced UNC5B throughout the glandular epithelial cells and apical surface and induction is much less in UNC5B^+/−^ mice. Netrin-1 staining restricted to basal surface of mucosa (Highlighted in yellow with red arrow point towards basal surface). No staining was seen in the apical surface. DSS induced large increase in netrin-1 staining throughout mucosa and apical surface. The induction of netrin-1 in UNC5B^+/−^ mice is much less as compared to WT mice colon with DSS.

UNC5B mediates cell survival signal in the presence of netrin-1 [9,14]. Netrin-1 expression was shown to be expressed in the base of the crypts and forced expression in apical surface suppresses cell death, which leads to the formation of colon polyps [15]. However, spatial regulation of netrin-1 and its receptors in colitis model are not clearly examined. We examined the hypothesis that in response to injury, the compartmentalized expression of netrin-1 and UNC5B is altered to provide survival signal. Consistent with our hypothesis, UNC5B expression is restricted to the apical surface of colon epithelial cells of the mucosa (Fig. 7 and Fig. S1). The intensity of staining drastically reduced in UNC5B^−/+^ mice colon epithelium, but apical expression is not changed. However, netrin-1 expression is restricted to basal surface of mucosa (in the goblet cells near submucosa) (Fig. 7). Netrin-1 expression is also reduced in UNC5B^−/+^ mice colon. DSS treatment increases the intensity of UNC5B staining throughout the mucosa both basal and apical surface especially in goblet cells. Again the induction is much less in UNC5B^−/+^ mice colon. Similarly, DSS treatment induced netrin-1 throughout the mucosa and intensity was increased towards apical surface (Fig. 7). However, netrin-1 induction is much less in UNC5B^−/+^ mice colon. Our results suggest that in response to injury, netrin-1 and UNC5B expression are induced in the same compartment which may enable UNC5B to transduce survival signals.

### Forced overexpression of UNC5B in human colon epithelial cells reduced DSS-induced apoptosis but increased apoptosis under basal condition

**Fig. 8:**
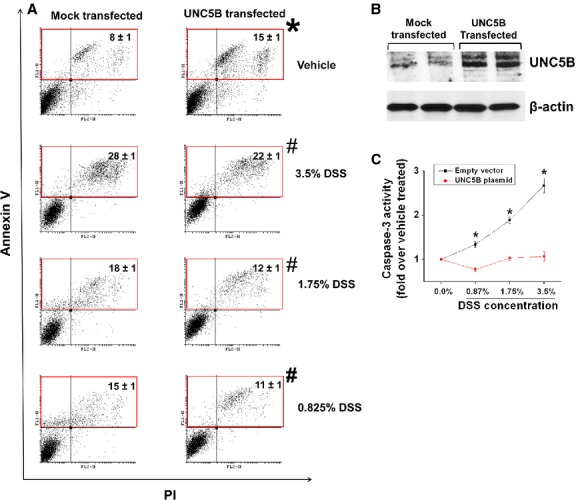
Overexpression of UNC5B receptor in human colon epithelial cells suppresses DSS-induced apoptosis and caspase-3 activity. (**A**) Human colon epithelial cell was transfected with rat UNC5B expression plasmid or empty vector. Forty-eight hours after transfection apoptosis was quantified using Annexin V and propidium iodide staining and labelled cells were analysed in flow cytometry. *P < 0.05 *versus* vehicle-treated vector transfected cells. ^#^*P* < 0.001 *versus* DSS-treated vector transfected cells; *N* = 5. (**B**) Overexpression of UNC5B was confirmed by Western blot analysis. (**C**) Caspase-3 Activity was quantified in transfected cells 24 hrs after DSS treatment as described in Materials and methods. *P < 0.05 *versus* corresponding UNC5B transfected cells; *N* = 5–8.

**Fig. 9:**
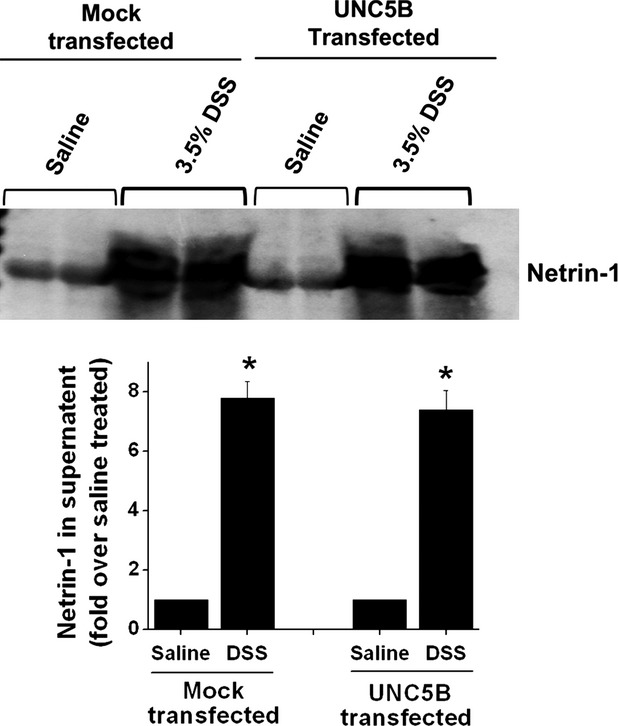
DSS treatment induced large amount of netrin-1 human colon epithelial cells. Colon epithelial cells were treated with 3.5% of DSS for 24 hrs and netrin-1 secretion into the supernatant was quantified by Western blot analysis followed by densitometric quantification of the bands.

To determine directly whether UNC5B mediates survival signal in response to injury, human colon epithelial cells were transfected with UNC5B expression construct or empty vector. Overexpression was confirmed by Western blot analysis (Fig. 8A). Then cells were treated with different concentration of DSS and apoptosis were determined at 24 hrs after treatment as described in Materials and methods. Interestingly, UNC5B overexpression alone increased apoptosis as compared to control (Fig. 8B). However, DSS-induced apoptosis was significantly suppressed in UNC5B transfected cells as compared to control suggesting UNC5B transmits survival signal in response to injury. Consistent with our view, netrin-1 expression was also strongly induced in response to DSS treatment both *in vivo* (Figs 1 and 7) and *in vitro* (Fig. 9).

### shRNA-based knockdown of netrin-1 expression exacerbates DSS-induced cell death in human colon epithelial cells

**Fig. 10:**
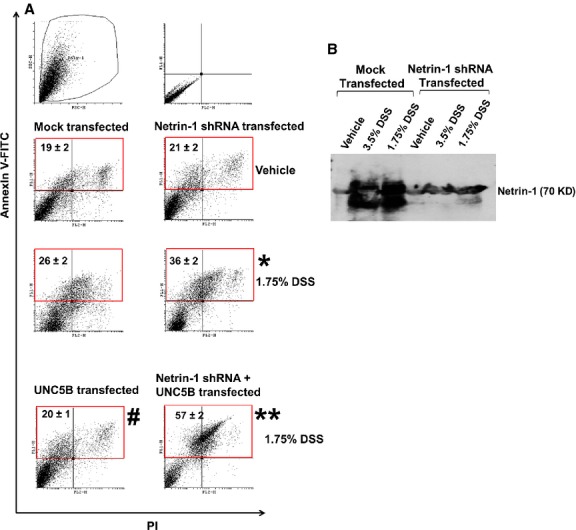
Suppression of netrin-1 expression using shRNA-based anti-sense strategy increased DSS-induced apoptosis and abolished UNC5B mediated protection of epithelial cell. (**A**) Human colon epithelial cell was transfected with shRNA expressing plasmid and/or rat UNC5B expression plasmid or empty vector. Forty-eight hours after transfection apoptosis was quantified using Annexin V and propidium iodide staining and labelled cells were analysed in flow cytometry. *P < 0.05 *versus* DSS-treated vector transfected cells. ^#^*P* < 0.05 *versus* DSS-treated vector transfected cells. **P < 0.001 *versus* UNC5B transfected DSS-treated cells; *N* = 5. (**B**) Western blot analysis of netrin-1 expression in colon epithelial cells supernatant with/without netrin-1 knockdown.

As netrin-1 is induced both *in vitro* and *in vivo*, we examined the hypothesis that netrin-1 induction was associated with cell survival and in the absence of netrin-1 expression the DSS-induced apoptosis will be exacerbated. As shown in Figure 10B, shRNA transfection reduced netrin-1 expression over 80%. Treatment of DSS to mock transfected epithelial cells induced a significant increase in apoptosis over vehicle treated. DSS-induced apoptosis was further increased in netrin-1 shRNA transfected cells suggesting netrin-1 expression is required for cell survival in response to stress.

To determine whether UNC5B mediated suppression of DSS-induced apoptosis, human colon epithelial cells were transfected with UNC5B overexpression of plasmid alone or with netrin-1 shRNA plasmid. As shown in Figure 10, in the absence of netrin-1 knockdown, UNC5B overexpression reduced apoptosis. However, when the netrin-1 expression was down-regulated with shRNA, UNC5B mediated suppression of apoptosis is not seen rather it increased apoptosis suggesting that UNC5B reduce apoptosis by binding to its ligand netrin-1 and activating cell survival pathways.

## Discussion

The functional role of guidance protein receptor UNC5B was described in the nervous systems related to development and axon guidance [16]. Subsequent studies had shown functions beyond the nervous system such as immune cell adhesion, migration, the orchestration of an acute inflammatory response [6], cancer cell growth and apoptosis [7,17], reperfusion injury of heart [18] and kidney [6] and angiogenesis [10]. Netrin-1 expression is always required to mediate UNC5B cell survival activity and in the absence of ligand, UNC5B receptor induced apoptosis. The name dependence receptor was coined to reflect UNC5B receptor dependence on the ligand for cell survival [8]. Several studies had shown that netrin-1 expression is up-regulated during tissue injury [3,19,20]. Therefore, the availability of ligand will not be a problem during stress. Therefore, we hypothesized that UNC5B signalling is critical for cell survival under stress. Thus, we investigated the role of UNC5B during DSS colitis. We report here that UNC5B^+/−^ mice showed exacerbation of colon injury by increasing epithelial cell apoptosis. In addition, UNC5B and netrin-1 expression is compartmentalized in normal colon, but DSS administration altered the compartmentalization, *i.e*. both netrin-1 and UNC5B expressed throughout colon mucosa. Partial deletion of UNC5B exacerbated DSS colitis suggesting protective function of UNC5B against colitis. This protective function may be mediated through suppression of apoptosis. Netrin-1 induction was reduced in the absence of UNC5B expression. Forced overexpression of UNC5B reduced apoptosis and caspase-3 activation. SiRNA mediated reduction in netrin-1 expression exacerbates DSS-induced apoptosis in colon epithelial cells. We do not see any difference in Ki67 positive cells in WT and UNC5B^+/−^ mice colon suggesting proliferation of epithelial is not significantly altered with partial deletion of UNC5B. As such, we provide evidence that UNC5B act as survival receptor during DSS-induced colitis and might serve as potential therapeutic target to prevent colon injury in the future.

UNC5B expression was found outside the nervous systems such as kidney, heart, lung, liver, colon, small intestine, spleen, blood, neutrophils, macrophages and lymphocytes [6,18,21–24]. This expression pattern suggests that UNC5B holds the function outside the CNS. Indeed UNC5B is implicated into different processes such as angio- [10] and cancerogenesis [7] as well as the control of acute inflammation [6] and ischaemia reperfusion injury [19,25]. Endothelial-specific deletion of UNC5B gene is lethal for embryogenesis because of defective angiogenesis [10]. During cancerogenesis, UNC5B is down-regulated in various kinds of tumour [7,26] affecting anchorage-independent growth and invasiveness and cell survival. Evidentially, UNC5B as dependence receptor mediates p-53-induced apoptosis by its death domain [27,28], which is cleaved off when netrin-1 is not bound to the UNC5B receptor. In the presence of netrin-1, p53 can be overexpressed but still did not induce apoptosis. Further analysis had shown that survival signal such as PI3 kinase/Akt activated by netrin-1 binding to UNC5B [29] plays a critical role in inactivating p53. More recent studies from our laboratoryhad shown that UNC5B deletion in kidney epithelial cells exacerbated tissue injury through increased apoptosis and p53 activation [25]. Consistent with these findings, UNC5B deletion also increases tissue injury through increasing apoptosis suggesting that UNC5B-mediated survival signal is required for protection of tissue from injury.

UNC5B overexpression in cells induced apoptosis where the availability of ligand is limited. Therefore, UNC5B is proposed to be a tumour suppressor. However, universality of this concept is not clear. UNC5B expression is widespread in many tissue and cell types [6,24]. However, extensive apoptosis or tissue injury is not seen. Moreover, forced overexpression of UNC5B in the kidney epithelial cells does not induce apoptosis [25] whereas overexpression in several cancer cells induced apoptosis [17,27,28,30]. Consistent with these results, our studies in colon epithelial cells show that forced overexpression of UNC5B increased apoptosis at basal condition, but suppressed apoptosis in response to injury. The reason for this opposing action of UNC5B in basal *versus* injury may be because of the ligand availability. Tissue or epithelial cell injury induced large amount of netrin-1 therefore it may act to suppress cell death by activating cell survival pathways. This view was further supported by our observation that knockdown of netrin-1 exacerbated apoptosis even in the presence of UNC5B overexpression in response to DSS treatment (Figure 10).

Netrin-1 and UNC5B expression in colon mucosa is compartmentalized (Fig. 7) in a non-overlapping manner suggesting that they may have survival *versus* apoptosis in that region. Netrin-1 expression is restricted to basal and crypt of the mucosa consistent with proposed role as cell survival and maintenance of stem cells. Surface epithelial cell constantly undergo apoptosis, which was replaced with new cells that migrate from the crypts. Therefore, in the absence of such balance, it may lead to accumulation of more surviving cells and formation of tumour. This view was supported by studies using transgenic overexpression of netrin-1 in the apical surface, which suppressed cell death and induced colon polyps in mice [15]. UNC5B expression is restricted to the apical surface where apoptosis and cell renewal are required. However, the compartmentalized expression is abolished in response to injury suggesting the need for an activation of UNC5B-mediated cell survival pathways. Our *in vitro* data support the *in vivo* data that netrin-1 is highly induced after injury which is required for cell survival. Interestingly, netrin-1 induction is blunted in the absence of UNC5B expression suggesting possible autoregulation of its ligand. The mechanism for such autoregulation is not clear.

In summary, our studies document for the first time that UNC5B receptor is a critical determinant of cell survival in a model of DSS-induced colitis. Netrin-1 and UNC5B expression is compartmentalized under normal conditions, which was altered in response to injury. Moreover, netrin-1 induction is required for UNC5B-mediated suppression of DSS-induced apoptosis. In conclusion, a therapy based on the activation of UNC5B-mediated cell survival pathway may help to treat inflammatory bowel disease.

## Supporting information

**Figure S1.** Immunohistochemical localization UNC5B receptor and netrin-1 in WT (UNC5B+/+) mice colon treated with water or DSS. **Figure S2.** Immunohistochemical localization of monocyte and macrophage in WT (UNC5B+/+) and heterozygous UNC5B knockout mice colon that are fed with water or DSS.
