# Supplementary material for: UNC5B receptor deletion exacerbates DSS-induced colitis in mice by increasing epithelial cell apoptosis
Source: J Cell Mol Med. 2014 Apr 10;18(7):1290–9. doi: 10.1111/jcmm.12280 (PMC4117732; doi:10.1111/jcmm.12280)
Supplement: Supplementary file 1 [file jcmm0018-1290-SD1.docx]

**UNC5B receptor deletion exacerbates DSS induced colitis in mice by increasing epithelial cell apoptosis**

Punithavathi Ranganathan^1^

Calpurnia Jayakumar^1^

Dean Y.Li^2^

Ganesan Ramesh^1^

^1^Vascular Biology Center

Georgia Regents University

Augusta, GA 30912

^2^Program In Molecular Medicine

University of Utah

Salt Lake City, UT 84112

**RUNNING TITLE**: UNC5B and Colitis

**Correspondence Address:**

Ganesan Ramesh, Ph.D.

Department of Medicine/Vascular Biology Center, CB-3702

Georgia Regents University

1459 Laney-Walker Blvd

Augusta, GA 30912

## TEL. NO. (706) 721-9728

FAX. NO. (706) 721-9799

E-mail: [gramesh@gru.edu](mailto:gramesh@gru.edu)

Supplementary Figure S1.


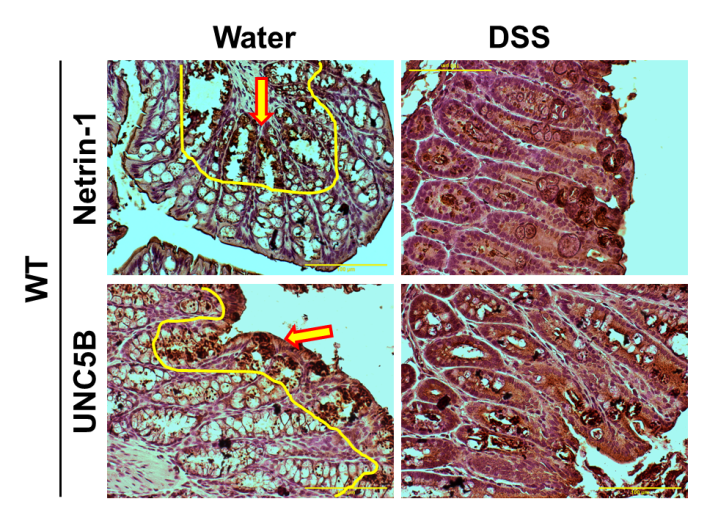


Figure S1. Immunhistochemical localization UNC5B receptor and netrin-1 in WT

(UNC5B+/+) mice colon treated with water or DSS. Representative higher magnification

(660X) picture is shown to depict the compartmentalization of netrin-1 and UNC5B

staining in the colon in water treated mice which was lost after injury with DSS.

UNC5B staining is restricted to apical epithelial cells of the mucosa (highlited in yellow

with arrow pointing toward apical surface). Netrin-1 staining restricted to basal surface

of mucosa (Highligted in yellow with red arrow point towards basal surface).

DSS induced large increase in netrin-1 and UNC5B staining throughout mucosa and

apical surface.

Supplementary Figure S2.


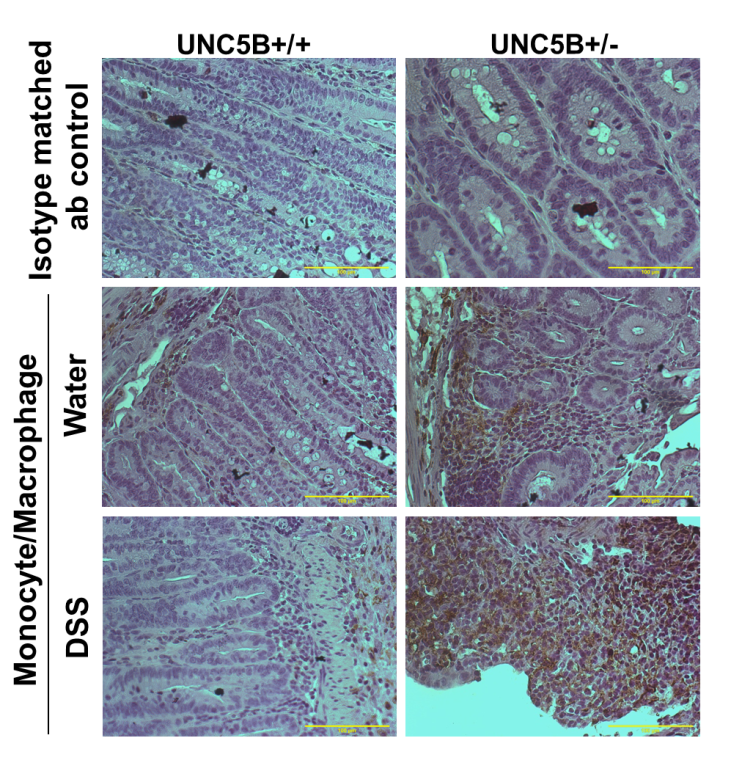


Figure S2. Immunohistochemical localization of monocyte and macrophage in WT

(UNC5B+/+) and heterozygous UNC5B knockout mice colon that are fed with water or

DSS. Monocyte staining was carried as described in Methods. A. No staining was seen

with isotype matched antibody controls. Very few cells were positive monocyte in water

treated UNC5B+/+ and UNC5B+/- colon. DSS treatment increased neutrophils influx as

seen by increased staining for monocytes in colon mucosa.
